# Anticardiolipin (aCL) in sera from periodontitis subjects activate Toll-like receptor 4 (TLR4)

**DOI:** 10.1371/journal.pone.0203494

**Published:** 2018-09-07

**Authors:** Harvey A. Schenkein, Ravindar R. Thomas

**Affiliations:** Department of Periodontics, Virginia Commonwealth University School of Dentistry, Richmond, Virginia, United States of America; University of the Pacific, UNITED STATES

## Abstract

Anticardiolipin antibodies (aCL) have been reported to be present in 15–20% of sera from subjects with periodontitis at concentrations exceeding those found in 95% of the healthy adult population. These antibodies, albeit at concentrations exceeding those generally found in periodontitis subjects, are typically present in patients with the antiphospholipid syndrome (APS), an autoimmune disease characterized by thrombosis and recurrent pregnancy loss. aCL from APS patients are proinflammatory and can activate trophoblasts, macrophages, and platelets via cell-surface interactions with their target antigen beta-2-glycoprotein-I (β2GPI). β2GPI is an anionic phospholipid-binding serum protein that can associate with toll-like receptors (TLR’s) on the cell-surface, leading to cell activation following interaction with autoimmune aCL. We examined an expanded series of 629 sera from clinically characterized subjects for aCL content, and observed that 14–19% of these sera contained elevated (>95^th^ %-tile) levels of aCL. We purified IgG from 16 subjects with elevated or normal levels of aCL and examined their ability to activate TLR2- or TLR4-transfected human embryonic kidney (HEK) cells, and observed that IgG from periodontitis patients with elevated aCL activated HEK-TLR4 cells, but not HEK-TLR2 cells. Prior removal of aCL by immunoabsorption significantly reduced the ability of IgG preparations from these sera to activate TLR4. Further experiments using a human first trimester trophoblastic cell line (HTR8 sv/neo) revealed that aCL from periodontitis patients stimulated IL-8 production, which was profoundly decreased if aCL was removed by immunoabsorption or if HTR8 sv/neo were pretreated with blocking anti-TLR4 antibodies. Thus, it appears that aCL from periodontitis patients can be proinflammatory, activating cells via TLR4. Since these antibodies are likely produced via molecular mimicry due to similarities between oral bacterial antigens and β2GPI, the data indicate that circulating serum aCL may induce or influence inflammatory responses at sites distant from the oral cavity.

## Introduction

Antiphospholipid autoantibodies (aPL), commonly found in sera of individuals with the antiphospholipid syndrome (APS) and systemic lupus erythematosus (SLE), are strongly associated with adverse pregnancy outcomes such as pregnancy loss and preeclampsia [1, 2]. Patients with APS also may have additional prothrombotic autoantibodies that predispose these individuals to venous and arterial thrombosis, stroke, and myocardial infarction [3, 4].

We have noted that a significant proportion of patients with aggressive and chronic periodontitis, approximately 15–20%, have serum levels of IgG or IgM anticardiolipin (aCL) in excess of those found in 95% of the healthy population [5]. Although the levels of aCL noted in periodontitis patients are mostly lower than those currently recommended in the classification criteria for diagnosis of APS [6], these antibodies have nevertheless been shown to have biological activity such as promotion of cytokine release from human vascular endothelial cells [7, 8]. Furthermore, such antibodies can be induced by immunization of animals with certain periodontal pathogens, including *Porphyromonas gingivalis*, *Aggregatibacter actinomycetemcomitans*, and *Treponema* denticola. This is likely an example of molecular mimicry, since all three pathogens have antigens with peptide sequences with significant homology to that of a critical antibody binding site in the serum protein β2GP1, the target antigen of aCL [9–11]. The anti-*P*. *gingivalis* antibodies raised in mice have biological activity leading to promotion of fetal loss in a mouse pregnancy model [9].

Among the many possible mechanisms whereby aCL autoantibodies might influence pregnancy outcomes is activation of macrophages, platelets and trophoblasts via toll-like receptors (TLR) [12]. There is some disagreement regarding the specific TLRs that can be activated by these autoantibodies. Various studies in patients with APS have demonstrated activation of TLR2, TLR4, or TLR8 by some aPL [13–18]. These divergent observations may be attributed to heterogeneity of these autoantibodies in APS patients. It has been proposed that TLR activation may occur due to cross-linking of β2GP1 by aCL, promoting activation of receptors that are associated with anionic phospholipids in lipid rafts [18]. Alternatively, it has been demonstrated that *P*. *gingivalis* bears an antigen with homology to peptide sequences found in both β2GP1 and TLR4, and thus antibodies reactive with *P*. *gingivalis* could directly bind to and activate TLR4 [19]. However, it has never been demonstrated that aCL found in sera from periodontitis patients can interact with and activate TLR4.

We hypothesized that IgG aCL from periodontitis patients could activate TLR4, thus explaining its ability to stimulate inflammatory cytokine release from trophoblasts and endothelial cells.

## Materials and methods

### Clinical methods and serum samples

This study was approved by the Internal Review Board of Virginia Commonwealth University. All subjects provided written informed consent, except in the case of minors, in which case written consent was obtained from parents or guardians, and subject assent was obtained as appropriate. Serum samples used for this study were from subjects described in previous publications [5, 7, 8]. The subjects included patients with generalized and localized aggressive periodontitis, chronic periodontitis as well as periodontally healthy individuals. All participants were determined to be systemically healthy by medical history. Specific exclusion criteria included a history of atherosclerosis (including coronary artery disease, cerebrovascular disease, or peripheral artery disease), diabetes mellitus, bleeding or thrombotic disorders, hepatic disease, systemic inflammatory or autoimmune diseases, uncontrolled hypertension, current chronic use of anti-inflammatory drugs, antibiotics, or statins, current pregnancy, and history of organ transplantation or immunosuppressive therapy.

Each participant received a comprehensive periodontal evaluation that included determination of pocket depth (PD), attachment loss (ALOSS), plaque index (PI) [20] and gingival index (GI)[21] at 6 sites per tooth. The examination protocol entails rounding of both ALOSS and PD measures down to the nearest lower whole number at each measured site. At the time of examination a blood sample was taken and processed for serum which was stored at -70°C. Serum samples chosen for inclusion in this study were selected from 629 samples previously assayed for aCl content (Table 1). The diagnostic criteria are as follows. Subjects with normal periodontium (NP) included those of any age with no evidence of AL or pockets greater than 3 mm, other than in buccal or lingual areas of gingival recession. Subjects with Chronic Periodontitis (CP) were of age >25 years with AL 2 mm or greater on multiple teeth in any extent or severity pattern consistent with plaque level and age. The definition of Generalized Aggressive Periodontitis (GAgP) for the subjects in this report conform to diagnostic criteria outlined by Armitage [22] with the additional requirement that such patients had a history of disease onset prior to age 35. They presented with at least 8 teeth with 5 mm or more attachment loss at interproximal sites, at least 3 of the affected teeth were not first molars and incisors.

**Table 1:** Clinical characteristics and serum anticardiolipin (aCL) levels for 629 subjects with or without periodontitis and for the subset of 16 subjects in this study.

Diagnosis	Mean ALOSS (mm±SE)	Mean PD (mm±SE)	Age (y±SE)	% female	IgG or IgM elevated^a^	% IgG or IgM elevated^b^
**Healthy**	0.27+.09	1.96+.05	34.6+.66	60.1	13/183	7.1
**CP**	1.90+.09	2.68+.05	44.3+.67	58.3	24/175	13.7
**LAgP**	0.79+.08	2.65+.06	24.2+.75	64.5	20/141	14.1
**GAgP**	3.10+.11	3.82+.06	30.1+.78	66.9	25/130	19.2
**Subset of 16 subjects in this study**						
**Healthy**	0.23+1.3	2.09+.46	33.5+8.7	60.0	0/5	0.0
**CP**	3.79+1.1	2.70+.42	54.6+7.7	60.0	4/5	80.0
**GAgP**	3.56+1.04	3.31+.38	37.5+7.1	60.0	2/6	33.3

^a^Elevated IgG aCL: >15 GPL/ml (95^th^ percentile = 5.7, 99^th^ percentile = 23.8); Elevated IgM aCL: > 15 MPL/ml (95^th^ percentile = 11.7, 99^th^ percentile = 29.9)

^b^Most of the periodontitis patients testing positive are in the “low positive” range of 15–40 GPL/ml or MPL/ml

Note: The Sydney Classification for APS diagnostic criteria specify that aCL levels >40 GPL or MPL on one occasion, or levels >99^th^ percentile determined on 2 or more occasions, are one of several criteria needed to conclude a diagnosis of APS.

### Purification of IgG and depletion of aCl from IgG preparations

Purification of IgG using Protein G Sepharose and quantification of IgG from subjects’ samples was accomplished as previously described in detail in [8]. Sera and IgG fractions were assayed for anti CL using the Varelisa Cardiolipin IgG or IgM kit. (Phadia, Thermo Fisher Scientific, US). Generation of an aCl immunoabsorbant was accomplished using the method of Pengo and Biasiolo [23]

### Cell culture and cytokine determination

Engineered Human Embryonic Kidney (HEK293) cell lines HEK-Blue ^TM^ hTLR2 and HEK-Blue ^TM^ hTLR4 were purchase from InvivoGen, USA, and utilized for determination of stimulation of human TLR2 or TLR4 respectively. The cells were grown and maintained according to manufacturer’s instructions. When cells were grown to 60–70% confluence, they were washed with PBS and detached with 3-5ml 1X PBS using a cell scraper. A cell suspension of ~300,000 cells/ml was prepared in warm HEK-Blue Detection medium. Approximately 20ul of each IgG sample (including samples from healthy controls, and periodontitis subjects with normal or elevated aCL) were placed in a 96 well sterile plate in replicate wells at 20ug/ml. 180ul of cells in HEK-Blue Detection medium was added to each well and the plate was incubated at 37°C in 5% CO2 for 16–18 hours. For determination of dose-responsiveness of HEK-Blue ^TM^ hTLR4 to IgG, serial dilution of 2.5, 5.0, 10 and 20ug/ml were prepared. All samples were examined in duplicate determinations.

The HTR8/SVneo trophoblast cell line was the kind gift of Dr. Charles H. Graham (Department of Anatomy & Cell Biology, Queen's University, Kingston, Ontario, Canada). It was grown in RPMI-1640 containing 5% FBS in 75 cm^2^ vented flasks at 37°C in 5% CO2. For determination of IL-8 production, about 200ul of HTR8/SV.neo cells were loaded in a 96 well plate at 300,000cells/ml and after 48 hours of incubation the supernatant was removed. 100ul of growth medium or neutralizing anti-hTLR4-IgG antibody (20ug/ml) (InvivoGen, USA) was loaded in replicate wells. After 1 hour of incubation at 37°C, 100ul of IgG or IgG depleted of aCL antibodies were loaded at 20ug/ml and incubated overnight. The cell supernatant was used to determine IL-8 using ‘Quantikine ^®^ELISA’ kits (R&D Systems) as described by the manufacturer.

### Statistical methods

Intergroup analyses were performed using ANOVA, with the Tukey HSD post-hoc test where appropriate.

## Results

We assessed concentrations of IgG and IgM aCL antibodies in sera from 629 adult subjects who were previously determined to be periodontal healthy or to have one of three subforms of periodontitis. As seen in Table 1, between 13.7% and 19.2% of subjects with periodontitis (chronic or aggressive) had serum IgG or IgM concentrations in excess of levels (15 GPL/ml or MPL/ml) found in 95% of the general healthy population. In our sample of subjects, about 7% of individuals who were systemically healthy by history and without periodontitis displayed aCL levels in excess of this concentration. For each periodontitis diagnosis, there was a significantly greater proportion of subjects with elevated levels of aCL compared to healthy controls. Sera for the remainder of the subsequent studies were randomly selected from 15 subjects with elevated or normal aCL serum concentrations. Table 1 further illustrates the mean whole mouth clinical measures of attachment loss and probing pocket depth for both all subjects in our database with aCL determinations and for the subset of subjects chosen for assessment of TLR activation. In addition, demographic data including mean age and proportion of female subjects is also provided.

**Fig 1:**
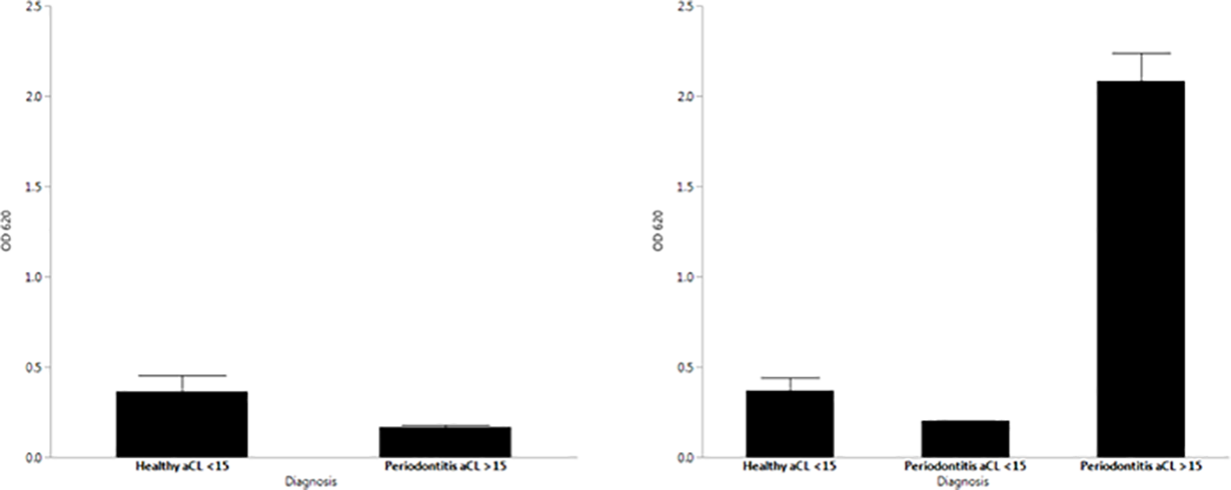
**Activation of HEK-Blue hTLR2 (left panel) and HEK-Blue hTLR4 (right panel) by IgG preparations from periodontally characterized subjects**. No difference in TLR2 activation was noted between healthy subjects with aCL<15 GPL/ml and periodontitis subjects with aCL>20 GLP/ml. (Student t-test). Activation of TLR4 by IgG from periodontitis subjects with aCL>20 GLP/ml was significantly greater than either IgG from healthy subjects or periodontitis subjects with GPL<15 GPL/ml (p < .005, ANOVA and Tukey’s HSD).

We examined the ability of IgG purified from 10 subjects’ sera to activate TLR2 using HEK-Blue-TLR2 cells. Five of these sera were from subjects with periodontitis, having aCL titers ranging from 20 to 267 GPL/ml. Four sera were from periodontally healthy subjects with aCL titers below 3.0 GPL/ml. As seen in Fig 1, there was minimal activation of TLR2 by these IgG preparations.

Next, we tested the same IgG preparations using HEK-Blue-TLR4 cells. As seen in Fig 1, IgG in sera from periodontitis subjects containing elevated aCL stimulated TLR4 activation to a much greater extent than did samples from periodontally healthy individuals. We also determined TLR4 activation by IgG from 5 periodontitis patients with normal levels of aCL, and observed that these samples failed to stimulate TLR4 to a greater extent than IgG samples from periodontally healthy controls.

**Fig 2:**
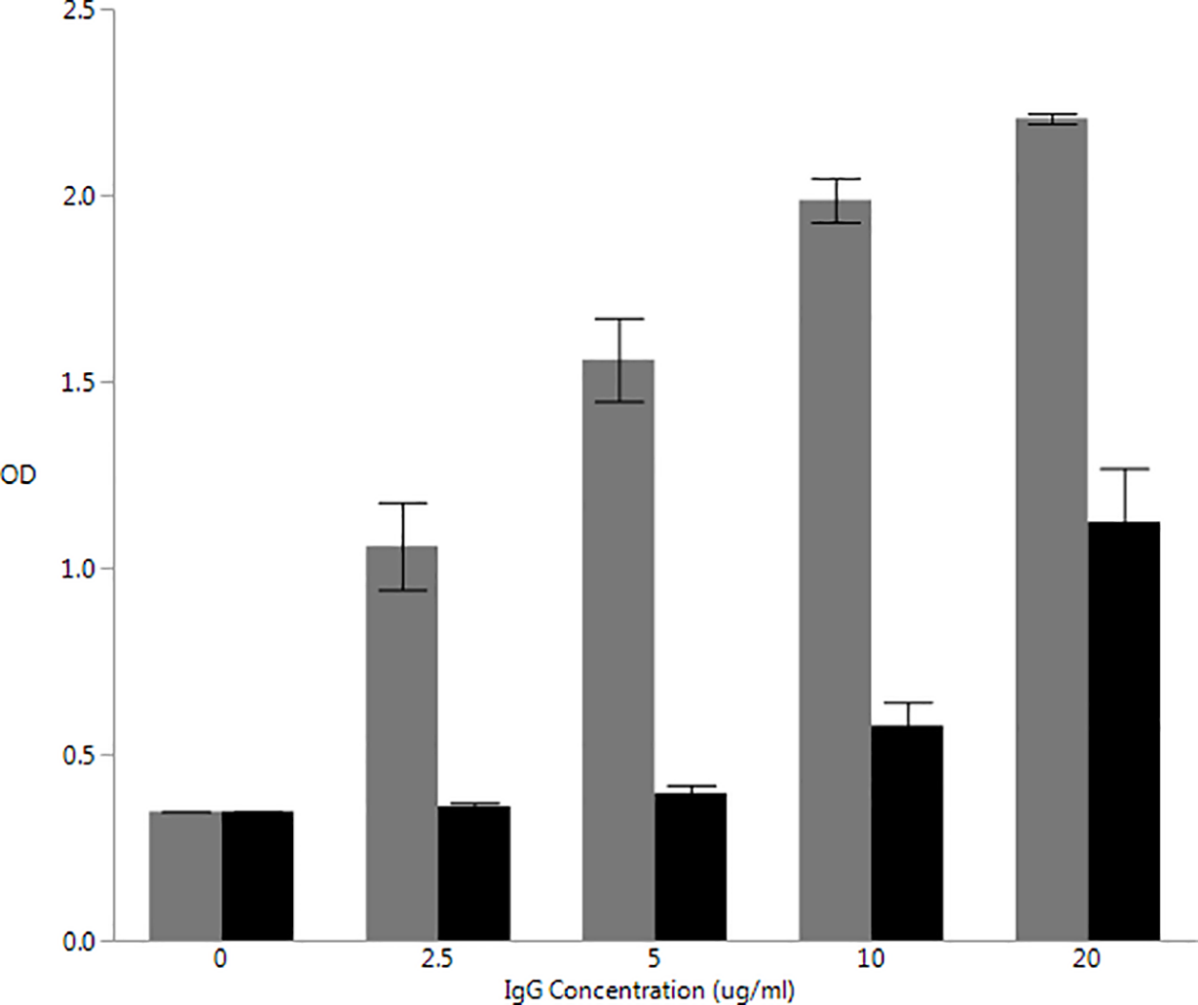
Activation of HEK-Blue hTLR4 by IgG purified from periodontitis sera before and after immunoabsorption to remove aCL. Activation of HEK-Blue hTLR4 by increasing concentrations of IgG derived from periodontitis patients with IgG aCL > 20 GPL/ml (grey), and effect of absorption of aCL from sera using cardiolipin-Sepharose on TLR4 activation (black). At each IgG concentration, significantly decreased stimulation by cardiolipin-Sepharose-treated samples was observed (p < .0001, ANOVA).

We have previously observed significant reduction of aCL titers, and reduced stimulation of MCP-1 production by human endothelial cells, following immunoabsorption on affinity columns bearing cardiolipin [8]. We chose 3 sera containing elevated aCL and processed them with one passage over a cardiolipin-containing affinity column, and compared TLR4 activation following purification of IgG. As seen in Fig 2, one passage over the affinity column to remove aCL antibodies reduced TLR4 activation significantly at all IgG concentrations tested (p = 0.0001). A small increase in TLR4 activation was noted at higher tested IgG concentrations (20 ug/ml), which was likely due to residual aCL or a minor contaminant such as LPS. Thus, TLR4 activation was due to serum antibodies similar or identical to aCL.

**Fig 3:**
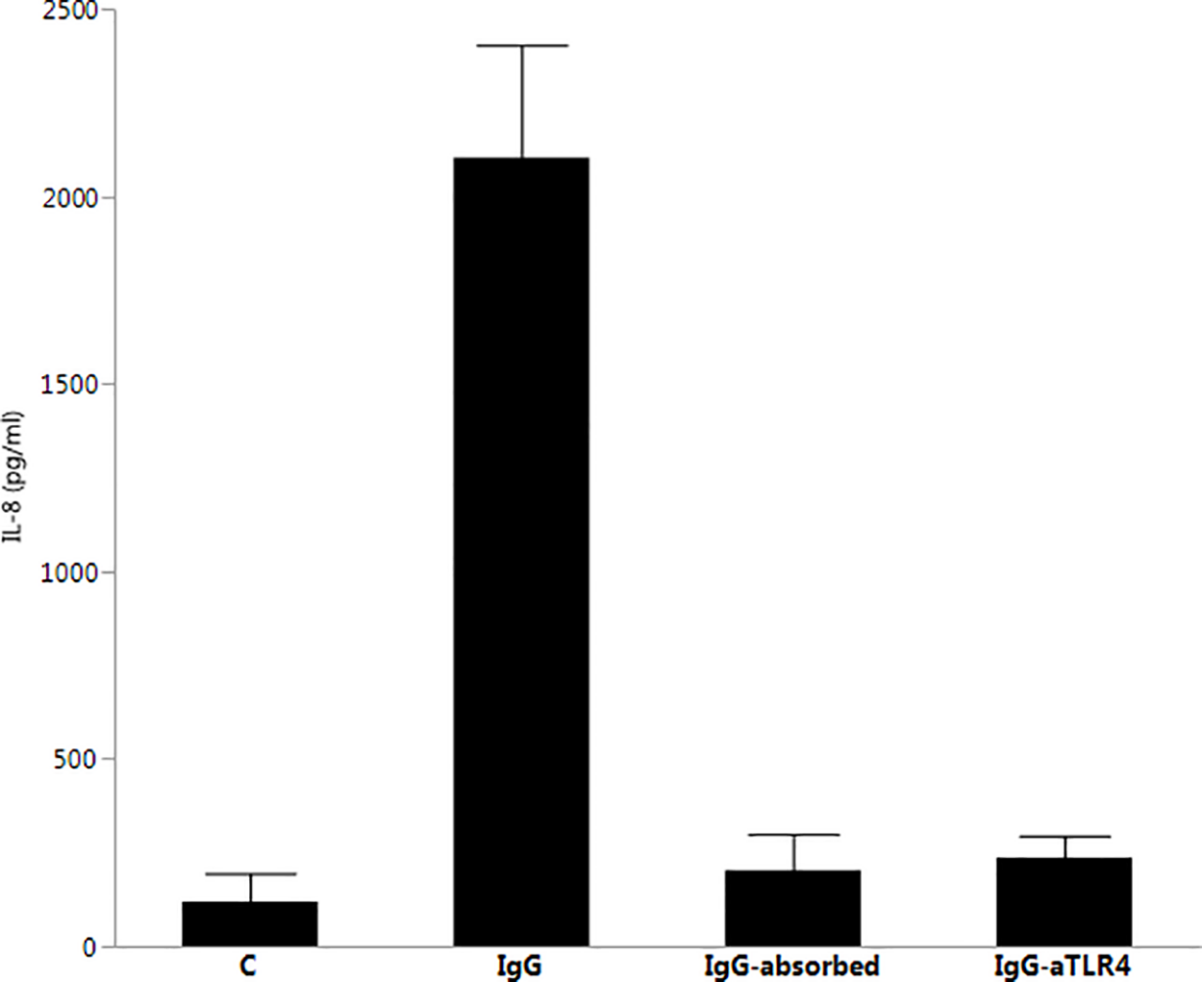
IgG-mediated stimulation of IL-8 production by HTR8/SV.neo (trophoblastic cell line), and inhibition by pre-treatment with mouse anti-hTLR4 or by absorption on cardiolipin-Sepharose. IL-8 stimulation by IgG preparations was significantly greater (p < .0001) than other groups (ANOVA and TUKEY HSD). C: unstimulated control; IgG: cells treated with 20 ug/ml purified IgG; IgG-absorbed: cells treated with IgG (20 ug/ml) purified from sera previously absorbed using cardiolipin-Sepharose; IgG-aTLR4: cells first incubated with anti-human TLR4 monoclonal antibody prior to incubation with 20 ug/ml purified IgG.

Next, we examined the ability of IgG from periodontitis subjects’ sera containing elevated levels of aCL to activate a first trimester human trophoblastic cell line (HTR8/SV.neo) via TLR4. We incubated HTR8/SV.neo cells with seropositive IgG preparations (20 ug/ml) from 5 periodontitis patients and observed increased IL-8 production compared to control (Fig 3). Removal of aCL antibodies from these sera by immunoabsorption prior to IgG purification reduced IL-8 secretion to baseline levels. Similarly, pretreatment of HTR8/SV.neo cells with mouse monoclonal anti-human TLR4 antibodies prior to stimulation resulted in profound inhibition of IL-8 production by these IgG preparations. We have also measured IL-6 production by these cells with similar results; its production is significantly and substantially (but not completely) inhibited by removal of aCL by immunoabsorption and by pretreating the cells with mouse anti-hTLR4 (data not shown).

## Discussion

A subset of sera from subjects with chronic and aggressive periodontitis contain greater than 15 GPL/ml or MPL/ml IgG or IgM aCL antibodies (Table 1). These levels are greater than those seen in 95% of the healthy population [24] but are not sufficiently high to meet current diagnostic criteria for APS, especially in consideration of the revised classification criteria (so-called Sydney Classification for APS) published in 2006 [6]. These criteria, revised from those proposed in 1999 (the Sapporo Classification for APS) [25] suggest multiple determinations of aCL greater than the 99^th^ percentile, measured several weeks apart, as part of the definitive diagnosis of APS. The levels of “elevated” aCL noted in Table 1 are therefore based on serum concentrations greater than 95% of the normal population; these patients are systemically healthy by history and do not display vascular thrombosis. It should be pointed out that health histories for the subjects in this study were determined using a comprehensive medical history questionnaire and so there could be unidentified underlying conditions in some of the subjects that would predispose to elevated serum aCL concentrations. Most of the aCL levels in periodontitis patients with “elevated” antibody concentrations fall between the 95^th^ and 99^th^ percentile. Nevertheless, these antibodies appear to display the proinflammatory properties described when tested at modest concentrations in tissue culture and in the mouse pregnancy model. Their possible role in human disease remains to be examined.

The concept that microbial infections can lead to production of pathogenic antiphospholipids has been advanced by several investigators [9, 26–29]. Blank and coworkers [30] identified a monoclonal anti-β2GP1 antibody that induced fetal involution in a mouse pregnancy model. The specificity of this antibody was determined to be a peptide sequence, TLRVYK, in β2GP1. They went on to note that a variety of bacterial, viral, and fungal pathogens displayed antigens with homology to TLRVYK and that antibodies raised against these pathogens also were capable of inducing fetal loss.

Many mechanisms have been proposed that could contribute to spontaneous fetal loss in APS, amongst which are placental inflammation due to cytokine production, neutrophil and macrophage recruitment, and complement activation [31]. Anticardiolipin from APS patients have, under a variety of in vitro culture conditions, been shown to activate TLRs including TLR2, TLR4, and TLR8 [13–18]. Inflammation, along with thrombosis and other alterations in trophoblast function including effects on invasiveness, fusion, and hormone production, are thought to contribute to injury to the placenta, growth restriction, and fetal loss. In view of the many pathologic mechanisms operant in APS, it is likely that multiple mechanisms may account for the pathology seen in this disease. It is also noteworthy that some, but not all, aCL antibodies from APS patients have been observed to activate TLR’s [18], highlighting the heterogeneity of these antibodies with respect to specificity in their interaction with β2GPI.

In view of data demonstrating associations between periodontitis and adverse pregnancy outcomes such as preterm birth, low birthweight, and preeclampsia[32–35], we were interested in examining periodontitis-associated aCL for the ability to activate TLR. We focused on TLR2 and TLR4 activation and noted that IgG from aCL-containing periodontitis sera activate TLR4 in tissue culture. This unique observation with respect to IgG in sera from individuals with periodontitis may suggest an alternative explanation for the associations found between periodontitis and pregnancy in some patients. Furthermore, this potential mechanism would also be consistent with the observation by several investigators that periodontal therapy administered during pregnancy fails to alter pregnancy outcome [36], since IgG levels do not decrease significantly over a period of just a few months, even with treatment [37]. Thus, periodontitis patients with chronic untreated disease and chronically elevated antibody levels to periodontal pathogens may be adversely affected by such antibodies during pregnancy. In fact, a retrospective analysis of our database, which contains birthweight data for children of mothers with periodontitis, indicates a strong relationship between adverse pregnancy outcomes (low birth weight and prematurity) and elevated maternal serum aCL levels that were measured several years following the children’s births (unpublished observation).

A trivial explanation for activation of TLR4 in protein preparations derived from human sera could be that levels of lipopolysaccharide in the IgG preparations are sufficiently high to activated cells via this receptor. Despite efforts to remove LPS from such preparations, trace levels of endotoxin can still remain following purification. However, the experimental data would strongly argue against activation of TLR4 by LPS contributing significantly to the results. First, despite the fact that IgG purified from all subjects had some trace levels of LPS, only those that contained aCL activated HEK-Blue-TLR4 cells despite identical purification procedures (Fig 1). Secondly, activation of HEK-Blue-TLR4 cells by sera from which IgG was purified following immunoabsorption of aCL resulted in reduction of TLR4 activation to background levels over a range of aCL concentrations (Fig 2), arguing that receptor activation was primarily due to aCL content rather than LPS contamination. Additionally, stimulation of IL-8 production by HTR8/SVneo cells following exposure to IgG aCL was significantly reduced following one passage of aCL-positive sera over cardiolipin-Sepharose. Several studies have indicated that trophoblastic cells, despite bearing TLR4 and responding to some but not all aCL via TLR4, do not generate a typical inflammatory response when treated with bacterial LPS [38, 39]. Furthermore, it has been reported by Gierman and coworkers that LPS fails to stimulate a cytokine response in HTR8/Svneo cells, with lack of either IL-6 or IL-8 production [40]. These data argue that aCL itself from periodontitis sera is capable of activating TLR4.

The source of antigens that could induce aCL in periodontitis has been examined. After recognition by several investigators that microbial pathogens can induce aCL by “molecular mimicry” due to the presence of peptide sequences with homology to a variety of microbial pathogens, we noted that there is homology between the arg-gingipain protease of *P*. *gingivalis* and the binding sequence for aCL in β2GPI [5]. Subsequently it was shown that *A*. *actinomycetemcomitans* and *T*. *denticola* also have peptide sequences similar to the TLRVYK hexapeptide recognized by several pathogens, and that antibodies raised against these peptides are mutually cross-reactive [11]. In studies examining the effects of antibodies raised against *P*. *gingivalis* in a mouse pregnancy model, we noted that anti-*P*. *gingivalis* IgG, but not antibodies raised against an arg-gingipain defective mutant of *P*. *gingivalis*, induced fetal loss. Removal of aCL by immunoabsorption significantly decreased the rate of fetal loss in this model, implicating aCL induced by *P*. *gingivalis* in this pathology [9]. We have shown that human aCL antibodies activate endothelial cells in culture, further demonstrating that these antibodies have significant biological activities [8].

The concept that aCL antibodies in sera from periodontitis patients, which are likely antibacterial, can be pathogenic and proinflammatory rather than protective, is not commonly considered. There is no precedent for serum IgG antibodies derived from periodontitis patients to directly activate TLRs. This observation raises questions regarding the function of the antibody response in periodontitis, as it is likely that three common periodontal pathogens have the capacity to induce aCL and, possibly, to be proinflammatory. The high levels of IgG antibacterial antibody in sera from periodontitis patients, as would be observed in most such individuals, could circulate to organs distant from the oral cavity and could induce inflammatory responses, and perhaps frank pathology, in these sites. So, it is certainly possible that systemic associations of some conditions with the presence of chronic or aggressive periodontitis could, in part, be mediated by such antibacterial antibodies.

## Conclusions

In conclusion, sera from 15–20% of individuals with periodontitis contain aCL antibodies at concentrations greater than those seen in 95% of the healthy population. IgG from those subjects with elevated aCL activate TLR4 and are capable of eliciting cytokine production by the human trophoblastic cells line HTR8/SVneo. The data are consistent with the interpretation that activation of TLR4 by this mechanism may contribute to systemic inflammation that has been observed in periodontitis, and may contribute to the observed associations between periodontal and systemic inflammatory conditions.

## Supporting information

S1 Table Supporting data for Table 1. Clinical Characteristics and serum anticardiolipin (aCL) levels for 629 subjects with or without periodontitis.
(XLSX)

S2 Table Supporting data for Fig 1: Activation of HEK-Blue hTLR2 and HEK-Blue hTLR4 by IgG preparations from periodontally characterized subjects. (XLSX)

S3 Table Supporting data for Fig 2: Activation of HEK-Blue hTLR4 by IgG purified from periodontitis sera before and after immunoabsorption to remove aCL. (XLSX)

S4 Table Supporting data for Fig 3: IgG-mediated stimulation of IL-8 production by HTR8/SV.neo (trophoblastic cell line). (XLSX)
